# GSK-3 Beta Does Not Stabilize Cryptochrome in the Circadian Clock of *Drosophila*

**DOI:** 10.1371/journal.pone.0146571

**Published:** 2016-01-07

**Authors:** Robin Fischer, Charlotte Helfrich-Förster, Nicolai Peschel

**Affiliations:** Neurobiology and Genetics, Biocenter, University of Würzburg, Würzburg, Germany; Karlsruhe Institute of Technology, GERMANY

## Abstract

Cryptochrome (CRY) is the primary photoreceptor of *Drosophila’s* circadian clock. It resets the circadian clock by promoting light-induced degradation of the clock protein Timeless (TIM) in the proteasome. Under constant light, the clock stops because TIM is absent, and the flies become arrhythmic. In addition to TIM degradation, light also induces CRY degradation. This depends on the interaction of CRY with several proteins such as the E3 ubiquitin ligases Jetlag (JET) and Ramshackle (BRWD3). However, CRY can seemingly also be stabilized by interaction with the kinase Shaggy (SGG), the GSK-3 beta fly orthologue. Consequently, flies with SGG overexpression in certain dorsal clock neurons are reported to remain rhythmic under constant light. We were interested in the interaction between CRY, Ramshackle and SGG and started to perform protein interaction studies in S2 cells. To our surprise, we were not able to replicate the results, that SGG overexpression does stabilize CRY, neither in S2 cells nor in the relevant clock neurons. SGG rather does the contrary. Furthermore, flies with SGG overexpression in the dorsal clock neurons became arrhythmic as did wild-type flies. Nevertheless, we could reproduce the published interaction of SGG with TIM, since flies with SGG overexpression in the lateral clock neurons shortened their free-running period. We conclude that SGG does not directly interact with CRY but rather with TIM. Furthermore we could demonstrate, that an unspecific antibody explains the observed stabilization effects on CRY.

## Introduction

The circadian clock is an ancient mechanism in almost every living being on this planet, including bacteria, plants and animals [1]. This sophisticated evolved machinery allows all organisms, to adapt to the daily 24 hours environmental changes on this planet–caused by the earth’s rotation round its own axis [2].

On the molecular level the clock consists of a transcriptionally regulated negative feedback loop. In *Drosophila*, the two clock proteins Period (PER) and Timeless (TIM) inhibit the transcription factors Clock (CLK) and Cycle (CYC). CYC and CLK act as transcriptional activators of *per* and *tim*. The negative feedback loop runs in about 150 neurons inside *Drosophila’s* brain [3]. The circadian clock persists under constant darkness conditions (DD) and has to be synchronized every day to the light-dark cycle. In *Drosophila* one of the most important circadian photoreceptors is the blue light sensitive protein Cryptochrome (CRY) [4, 5]. This photopigment changes its conformation upon light reception, allowing CRY to bind to TIM and subsequently leading to phosphorylation and ubiquitination of this clock protein. Afterwards the proteasomal degradation of TIM is triggered [6, 7]. Consequently, the light induced degradation of the core clock protein TIM leads to a non-functional circadian clock under constant light exposure (LL) and the fruit fly loses its rhythmic behaviour. The loss of CRY on the other hand results in severe changes for *Drosophila* to adapt to a new light regime [4]. Without CRY, the flies remain rhythmic under LL conditions, because TIM is not permanently degraded anymore [8].

The light sensitivity of *Drosophila’s* clock and the degradation of TIM are dependent on several other factors. It was for example shown, that distinct TIM isoforms perform differently in the light–the longer TIM isoform has a reduced affinity towards CRY, leading to a more stable TIM protein in light conditions [9]. The E3 ubiquitin ligase Jetlag (JET) is another important factor, because JET induced ubiquitination of the phosphorylated TIM protein leads to TIM’s degradation in light [10, 11]. A current model predicts that the light activated CRY among others binds to JET, TIM and to another F-Box protein called Ramshackle (BRWD3) [12]. The preferred CRY target is the highly phosphorylated form of TIM. This conformation of the protein is mostly found in the cell’s nucleus. Ubiquitination and degradation in the proteasome follows the binding of CRY and TIM. CRY itself is very likely phosphorylated and ubiquitinated in light as well, to a larger extend by Ramshackle (BRWD3) and to a smaller by JET [12–14]. Other proteins might be involved in this process of phosphorylation and/or degradation as well. To name a few: Kismet, Quasimodo or the Cop9 Signalosome [15–17]. The phosphorylation of CRY and TIM argues for participation of kinases and phosphatases. It was shown, that several proteins phosphorylate TIM, i.a. Casein Kinase 2 (CK2) and GSK-3 beta [18–20].

It was already shown, that GSK-3 beta–in *Drosophila* called Shaggy (SGG)—is involved in this process [18, 21]. Overexpression of SGG in clock neurons leads to a dramatic change in the behaviour of a fly under constant darkness conditions (DD). SGG phosphorylates TIM protein, leading to an earlier entry of TIM into the nucleus and subsequently to a shortened period of about 20 hrs [18]. Interestingly another publication demonstrated that SGG stabilizes CRY dramatically–even under constant illumination—leading to a behavioural phenotype under LL as well [21]. When SGG is overexpressed in the clock neurons, especially in the Dorsal ones, the fly keeps its rhythmicity in light [21].

The aim of the present study was to investigate the phosphorylation and degradation of TIM and CRY under light conditions. We were especially interested in investigating the theory, why a stabilization of the photoreceptor CRY leads to less light-sensitive animals, particularly in LL. Furthermore we wanted to closer investigate the interaction of the different proteins in order to understand the responses of the molecular clock to light. In particular we aimed to understand the antagonistic roles of SGG and JET/Ramshackle on CRY’s stability in LL. JET is of particular interest, because we found earlier that JET weakens CRY stability in LL [13]. Thus, JET and SGG may work antagonistically on CRY. Unfortunately, we could not repeat the stabilizing effect of SGG on CRY.

## Results

### CRY stabilization in vitro

Co-expression of SGG in S2 cells was reported to result in a strong stabilization of CRY–even under constant light (LL) conditions [21], whereas JET makes CRY less stable, when the two proteins are co-expressed under the same conditions in LL [13]. Thus, we wanted to know whether co-expression of JET in the presence of SGG would diminish the stabilizing effect of SGG on CRY.

**Fig 1:**
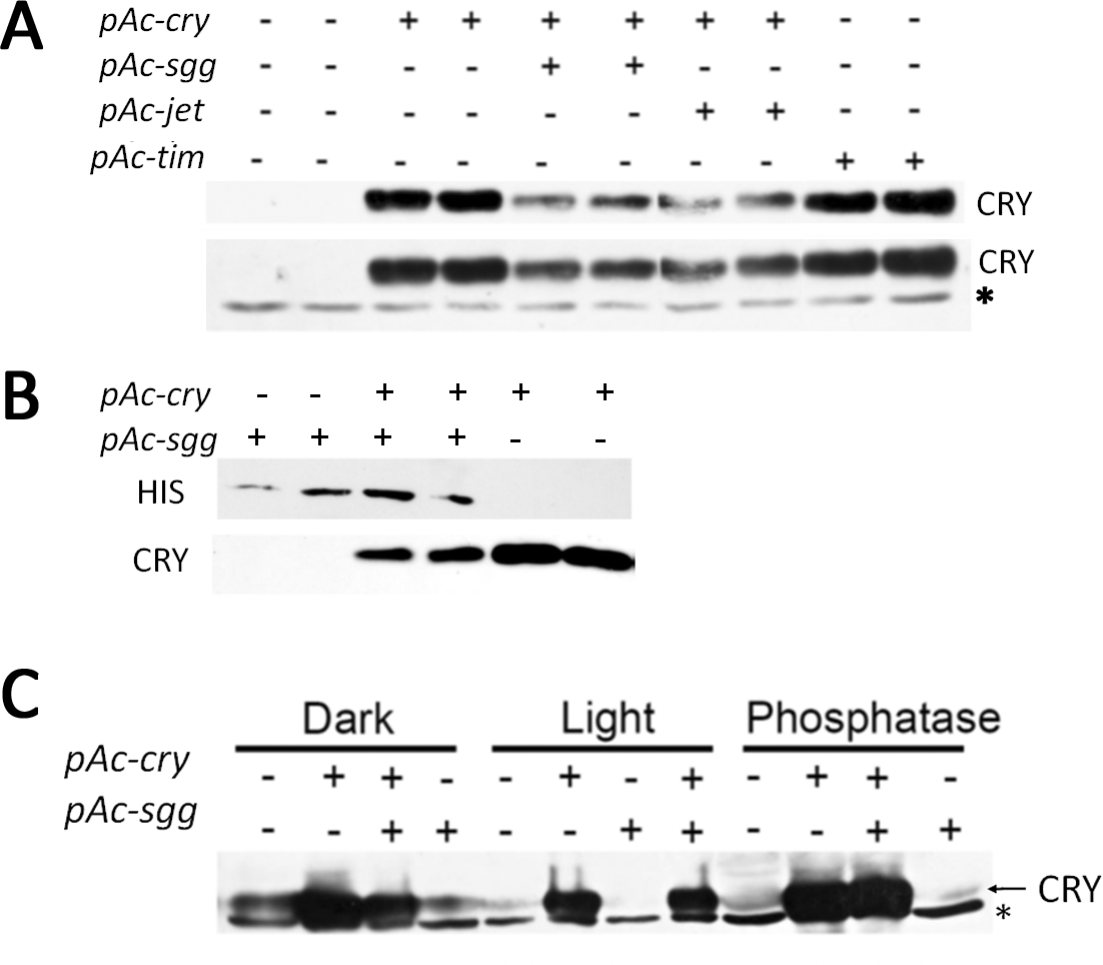
Stability of CRY in *Drosophila* S2 cells. *Drosophila* Schneider S2 cells were used to overexpress different genes under the control of a strong actin promoter. The transfected plasmids are indicated at the top and left, while (+) is transfected, (-) indicates no transfection. If not indicated otherwise the cells were kept in darkness. The antibody used to detect CRY Protein is described in Yoshii et al. 2008 [24]. (A) Representative blot of CRY stability in the presence of SGG, JET or TIM. The lower graph shows a longer exposed version of the blot. Here a non specific band(*) is visible to prove equal loading (B) Representative blot of SGG-HIS expression in S2 cells. The antibody used to detect the SGG-HIS protein was anti-HIS (Invitrogen). (C) This western blot was strongly overexposed to visualize the only weakly expressed endogenous CRY. Cells were either sacrificed in darkness, in light or the protein was treated with a λ-phosphatase for 1 hour (after being in dark). The asterisk marks a non-specific band.

But we were not able to see the stabilization effect of SGG on CRY (Fig 1A), we were even able to see a slight reduction in CRY under those conditions. *Drosophila* S2 cells were transfected with cDNA of several genes under the control of a strong actin promoter. The cells were kept in darkness and were only exposed to light for a very short time, while we harvested the cells and extracted the protein. When *jet* was expressed in S2 cells, we observed a small reduction in CRY level. On the other hand, we determined a small stabilization effect, when *tim* was co-expressed (as previously reported by us and others) [13, 22]. Because the positive and negative control worked well, we wondered why SGG expression does not stabilize CRY. To verify that the *pAc-sgg* plasmid (a generous gift from Pipat Nawathean) [21] was the correct one, we partially sequenced the plasmid and affirmed thereby that it was the proper plasmid (data not shown). Several repetitions of similar experiments yielded comparable results (data not shown); i.e., co-transfection and expression of *sgg* did not result in increased CRY levels. To confirm SGG expression in our S2 cell culture system we performed western blots using antibodies against the HIS-tag (Materials and Methods, Fig 1B). In the *pAc-sgg* plasmid, *sgg* is directly fused to a V5 and HIS-tag.

CRY is expressed endogenously in S2 cells in a very low level [23]. It was previously reported that this very low level of CRY protein in S2 cells can be stabilized dramatically (even under illumination) by co-expressing SGG [21]. In our hands, co-expression of SGG did not stabilize the endogenous CRY protein neither in dark nor in light (Fig 1C). Since SGG is a serine/threonine kinase, we were wondering if CRY’s phosphorylation status is changed by the co-expression of *sgg*. If our CRY antibody is not able to detect the postulated highly phosphorylated, slow migrating, form of CRY, we would not be able to see this protein on western blots. Therefore, we dephosphorylated CRY. After phosphatase treatment, we were not able to see a difference on western blots (Fig 1C). Although we have to stress at this point that the final proof showing that CRY is phosphorylated at all is still missing. We concluded that endogenous CRY is not stabilized in darkness or illumination and that CRY phosphorylation is not dramatically changed in the presence of SGG kinase.

### CRY stabilization *in vivo*

**Fig 2:**
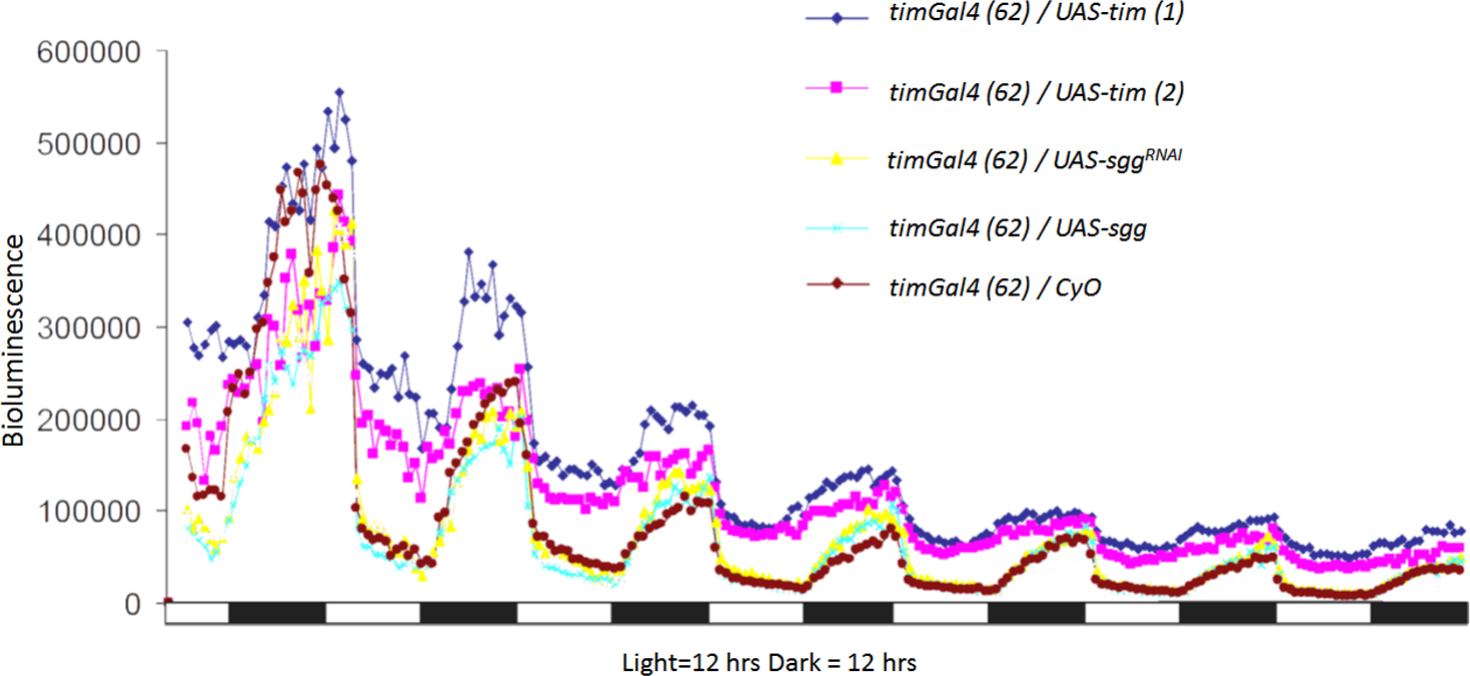
Stability of CRY in living *Drosophila* animals. Bioluminescence of adult *Drosophila* animals carrying a *Luc-dCry* reporter. Adult *Drosophila* transgenic male flies were measured in a Packard Topcount machine. The genotype of the flies is displayed on the right. Furthermore all animals carried one chromosomal copy of an *UAS*-*Luc-dCry* insertion. The x-axis indicates the time, the black and white bars at the bottom indicate the daily change of light and darkness (Light/Dark ratio 12:12 hrs). The y-axis indicates the bioluminescence level (in Counts per second CPS) and thus the luciferase amount. Per genotype 8 animals were investigated.

Next, we investigated the potential stabilization effect of SGG on CRY in adult *Drosophila* animals. Here we used a realtime luciferase assay to display the degradation of CRY under light/dark (LD) conditions [13, 25]. Therefore, we investigated flies carrying an *UAS*-*Luc-dCry* construct and a *timeless* driver (*tim-*Gal4) line to express the luciferase fusion protein in all clock cells. In those animals a luciferase protein, directly fused to CRY is expressed in all TIM positive cells, therefore the luciferase signal should reflect the CRY amount in all clock cells. Because we (and others) could already show that CRY is stabilized, when TIM is co-expressed [13, 22], we used two different *UAS*-*tim* lines as positive controls. To see whether SGG influences CRY stability we overexpressed or down-regulated *sgg* expression using *UAS-sgg* or *UAS-sgg*^*RNAi*^, respectively. The flies were kept in a luciferase plate reader for 7 days under L/D 25°C conditions. Consistent with our current and previous [13] S2 cell culture results, we found that only TIM overexpression stabilized CRY. Flies with SGG manipulation did not differ from the control. (Fig 2)

### Behaviour of SGG manipulated animals

Animals overexpressing SGG in clock neurons display a shortened period under DD conditions. Additionally they show morning and evening peaks under LD, which are dramatically shifted towards the midday/midnight [18]. It has also been reported that *sgg* over-expression in all or dorsal subsets of the clock neurons leads to rhythmic behaviour in LL, with ~50% (all neurons) or >90% (dorsal neurons) of the flies exhibiting robust rhythmicity [21]. Why a stabilization of CRY should make animals less sensitive towards light is not explained in detail. To get deeper insight we used two different fly strains for our overexpressing assay. One strain was carrying an *UAS*-*sgg* construct on the second chromosome, i.e. *P{UAS-sgg*.*B} MB5*, and another one that was previously used to demonstrate the behavioural effects of *sgg* expression in LL (*P{EP}sggEP1576*) [18, 21]. This strain contained an UAS insertion in the *sgg* region that could be addressed by the GAL4 protein to overexpress SGG. We included *P{UAS-sgg*.*B} MB5*, because we and others [18, 26] realized that this overexpression construct is much stronger than the original (*P{EP}sggEP1576*). Consistently we expected to see an even stronger phenotype with those animals. We used different driver lines to express *sgg* in several subsets of clock neurons in the *Drosophila* brain. While *tim*-Gal4 should address all clock neurons, *cry*-Gal4 is expressed mainly in the *cry* expressing cells [24]. If the influence of SGG on CRY is the reason for the changed behaviour of those animals in LL, we would expect so see the biggest effect with these drivers. The reason for this expectation is, that CRY is expressed especially in cells that are addressed by those driver lines. *cry*-Gal4/*pdf*-Gal80 and *tim*-Gal4,*pdf*-Gal80 animals only overexpress SGG in the dorsally located cell groups, while the *Clk 4-1M* Gal4 only expresses in a subgroup of the dorsal neurons [27] (further explanation in S1 Fig). Moreover, we included animals, where the *sgg* mRNA was knocked down by RNAi, to see if those animals behave very sensitive towards light. Furthermore flies overexpressing the clock gene *per* are also behaviourally more rhythmic under constant light [21, 28]. Thus, as another control, we included *UAS-per* animals as well.

**Fig 3:**
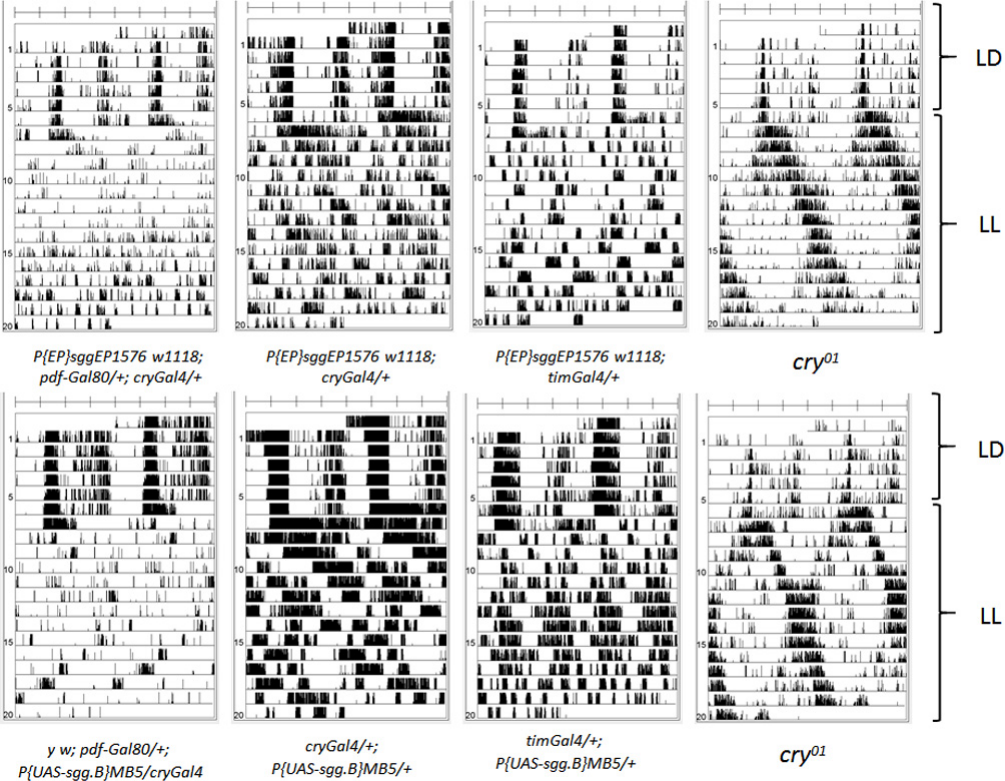
Rhythmic behaviour of male *Drosophila* flies in LL after SGG overexpression. Double-plotted actograms of representative single males of the rhythmic genotypes are shown. On the y-axis the LD or LL setting is marked. While the SGG overexpressing animals only show a very weak rhythm in LL, *cry*^*01*^ animals still behave strongly rhythmic under these conditions.

As expected, in DD overexpression of SGG in the ventral neurons shortened the period dramatically, especially when we used the *tim* driver line (Table 1) [18]. The only exception was the *P{EP}sggEP1576 w*^*1118*^*; cry-Gal4/+* line. A reason for the lack of phenotype might be, that the *cry*-Gal4 driver is not expressed as strong as the *tim*-Gal4 driver in the s-LNvs–the neurons that are most important for driving rhythmic behaviour in DD (an effect seen by others as well [29]). On the other hand the stronger effector line, *P{UAS-sgg*.*B} MB5* led to a short period of about 21 hrs in combination with the *cry*-Gal4 driver line demonstrating that the driver line is functional. The crossing of the combination of *P{UAS-sgg*.*B} MB5* and the strong *tim*-Gal4,*pdf*-Gal80 line did not yield in living offspring. In addition to the shortening or lengthening of the period in *sgg* manipulated animals a reduction in overall rhythmicity was observable. When we addressed different subsets of the dorsal neurons (*cry*-Gal4/*pdf-*Gal80 or *clk 4-1M* Gal4) we did not see an effect. While *cry*-Gal4/*pdf-*Gal80 addressed the *cry* expressing LNds and DN1s [30], *clk 4-1M* Gal4 is expressed in the DN1p only [27] (S1 Fig). Down-regulation of SGG resulted in long periods under DD conditions–again we did not see an effect, when we only down regulated SGG in the dorsal neurons (cry-Gal4/pdf-Gal80 or clk 4-1M Gal4) (Table 1). A similar difference was detectable, when we investigated the LD behaviour of the animals (S2 Fig). Those RNAi experiments clearly strengthen the direct influence of SGG on the clock (as shown by Martinek et al.) [18]. Our conclusion of those behaviour experiments is that *sgg* is only important in the lateral Neurons, especially in the *pdf* expressing small and large LNvs, but not in the dorsal Neurons (cry-Gal4/pdf-Gal80 or clk 4-1M Gal4)–at least for LD and DD behaviour. Strikingly, in our hands over-expression of *sgg* in the entire or parts of clock network did not elicit strong behavioural rhythmicity in LL. We investigated the behaviour in constant light with the same animals as in DD. Because the intensity of the light plays a very important role for the behaviour, we performed the LL experiments under three different light intensities, i.e. 50 lux, 300 lux and 1500 lux. The high light intensity condition was used in Stoleru et al. (2007) [21] so we expected to see a high number of rhythmic animals under LL conditions. While *cry* mutants showed the expected robustly rhythmic behaviour in LL, both *UAS-sgg* failed to produce significant LL rhythmicity when driven by the various clock-neuronal Gal4 lines (Table 2 and S1 Table). We did not observe a dramatic difference between the three light conditions, therefore we merged the data. In low light conditions we saw a general trend, of animals being more rhythmic in LL (S1 Table). This effect was especially pronounced in the two *pdf*-Gal80 driver lines, *cry*-Gal4/*pdf-*Gal80 and *tim-*Gal4,*pdf*-Gal80. But this higher number of rhythmic animals was mainly due to the driver line–the driver controls are almost as rhythmic as the *sgg* manipulated animals–and therefore the rhythm originates more likely from genetic background or darker eye colour, than from real *sgg* manipulation. The detected rhythms in all animals, except *cry*^*01*^, were very weak and faint (for power levels of the rhythms see S1 Table). In Fig 3 we compiled examples showing the rhythms observed after SGG overexpression in comparison to *cry*^*01*^. In contrast to *cry*^*01*^ mutants, almost none of the animals showed an obvious rhythm, which fits to the results of the periodogram analysis. Hence, the LL rhythm in those animals is almost, if not at all imperceptible. We performed a chi-square test to clarify if *sgg* manipulated animals differ from the control and did not find a significant difference (Chi^2^ from 0,15 to 3,76 and p ranging from 0,07 to 0,93) In total, we were never able to see such a dramatic LL effect, as did Stoleru et al. [21] The animals in which we overexpressed the *period* gene displayed a significantly stronger rhythm than the *sgg* manipulated animals (Chi^2^ from 11,4 to 14,9 and p ranging from 0,003 to 0,0006)–though the number and power of the rhythm was never as high as in *cry*^*01*^ (Chi^2^ 124,06 and p = 8,2 E-29) and as reported in the original publication [28]. An explanation for the difference in rhythmicity of *period* overexpressing animals compared to the original publication can be that we used a different UAS-*per* construct than Murad et al. [28] or Stoleru et al. [21]. When *per* was expressed in the Dorsal neurons only (cry-Gal4/pdf-Gal80 or tim-Gal4/pdf-Gal80), we found a stronger rhythm in those animals, compared to the control or the dorsal clk 4-1M Gal4 driver line.

**Table 1:** Behaviour in LD/DD conditions.

Group	Genotype	n	period	sem	Rhythmic %
Over expression of *sgg*	*P{EP}sggEP1576 w*^*1118*^*; Pdf-*Gal80*/+; cry-*Gal4*/+*	32	23,70	0,08	97
	*P{EP}sggEP1576 w*^*1118*^*; cry-*Gal4*/+*	27	24,91	0,18	91
	*P{EP}sggEP1576 w*^*1118*^*; tim-*Gal4*/+*	29	21,37	0,36	76
	*P{EP}sggEP1576 w*^*1118*^*;; Clk 4-1M* Gal4*/+*	16	24,38	0,06	100
	*P{EP}sggEP1576 w*^*1118*^*;tim-*Gal4,*pdf-*Gal80*/+*	14	24,83	0,13	89
Over expression of *sgg* (Uas)	*y w; Pdf-* Gal80*/+; P{*UAS*-sgg*.*B}MB5/cry-*Gal*4*	22	23,72	0,14	83
	*cry-*Gal4*/+;P{*UAS*-sgg*.*B}MB5/+*	29	20,94	0,14	77
	*tim-*Gal4*/+; P{*UAS*-sgg*.*B}MB5/+*	21	18,55	0,62	61
	*P{*UAS*-sgg*.*B}MB5/ Clk 4-1M* Gal4	16	24,16	0,07	100
Down regulation of *sgg* (RNAi)	*Pdf-*Gal80*/+; P{TRiP*.*GL00277 sgg RNAi}attP2/cry-*Gal4	29	24,20	0,14	93
	*cry-*Gal4*/+;P{TRiP*.*GL00277 sgg RNAi}attP2/+*	28	26,21	0,15	92
	*tim-*Gal4*/+; P{TRiP*.*GL00277 sgg RNAi}attP2/+*	31	27,03	0,14	100
	*P{TRiP*.*GL00277 sgg RNAi}attP2/Clk 4-1M* Gal4	16	24,36	0,23	100
	*P{TRiP*.*GL00277 sgg RNAi}attP2;35364xtim-*Gal4,*pdf-* Gal80*/+*	16	25,29	0,13	56
Over expression of *per*	*w*;*pdf*Gal80; UAS-*per16/*cry-Gal4	13	24,18	0,04	77
	*w*;cry-Gal4/+;UAS-*per16*/+	14	23,90	0,07	57
	*w;tim*-Gal4/+; UAS-*per16*/+	13	26,07	0,10	100
	*w;;*UAS-*per16*/ *Clk 4-1M* Gal4	13	24,64	0,10	92
	w;*tim-*Gal4,*pdf*-Gal80/+;UAS-*per16/+*	14	24,76	0,10	57
Control animals	*w*^*1118*^*; pdf-* Gal80*/+;cry-*Gal4*/+*	29	23,66	0,12	97
	*w*^*1118*^*; cry-*Gal4*/+*	28	24,69	0,17	92
	*w*^*1118*^*; tim-*Gal4*/+*	29	23,84	0,11	97
	*w*^*1118*^*;; Clk 4-1M* Gal4*/+*	32	23,90	0,11	97
	*w*^*1118*^*; tim-*Gal4,*pdf-* Gal80*/+*	14	23,61	0,17	71
	*P{EP}sggEP1576*, *w*^*1118*^	27	24,09	0,11	87
	*w*^*1118*^*;;UAS-per16/+*	13	23,63	0,08	92
	*w*^*1118*^*;;P{UAS-sgg*.*B}MB5/+*	30	23,47	0,13	93
	*w*^*1118*^*;; P{TRiP*.*GL00277 sgg RNAi}attP2/+*	31	23,52	0,15	90
Positive Control	*cry*^*01*^	29	23,07	0,08	97
	*w*^*1118*^	31	23,78	0,09	93

Flies (only males) were recorded in LD 12:12 for 7 days and subsequently in DD for at least 14 days. The table displays the percentage of rhythmic flies, the period length and the rhythm of all investigated genotypes in DD according to χ2-periodogram analysis. Furthermore, the number of investigated animals is indicated. Animals that died before the end of the experiment were excluded. For clarity reasons we did not included the power of the rhythmicity and the genetic background of the animals. This information can be found in Supplementary S1 Table and in S1 Fig.

**Table 2:** Behaviour in LD/LL conditions.

Group	Genotype	n	period	sem	Percent %
Over expression of *sgg*	*P{EP}sggEP1576 w*^*1118*^*; Pdf-*Gal80*/+; cry-*Gal4*/+*	120	27,30	1,06	15,21
	*P{EP}sggEP1576 w*^*1118*^*; cry-*Gal4*/+*	124	25,97	2,19	15,66
	*P{EP}sggEP1576 w*^*1118*^*; tim-*Gal4*/+*	116	26,14	1,19	22,39
	*P{EP}sggEP1576 w*^*1118*^*;; Clk 4-1M* Gal4*/+*	48	25,80	0,91	10,69
	*P{EP}sggEP1576 w*^*1118*^*;tim-*Gal4,*pdf-*Gal80*/+*	123	23,30	1,04	26,71
Over expression of *sgg* (Uas)	*y w; Pdf-* Gal80*/+; P{*UAS*-sgg*.*B}MB5/cry-*Gal*4*	98	24,47	2,48	30,01
	*cry-*Gal4*/+;P{*UAS*-sgg*.*B}MB5/+*	115	25,37	1,63	30,52
	*tim-*Gal4*/+; P{*UAS*-sgg*.*B}MB5/+*	104	26,96	1,65	6,51
	*P{*UAS*-sgg*.*B}MB5/ Clk 4-1M* Gal4	48	7,77	0	2,22
Down regulation of *sgg* (RNAi)	*Pdf-*Gal80*/+; P{TRiP*.*GL00277 sgg RNAi}attP2/cry-*Gal4	112	25,67	1,22	15,94
	*cry-*Gal4*/+;P{TRiP*.*GL00277 sgg RNAi}attP2/+*	125	25,31	1,47	16,30
	*tim-*Gal4*/+; P{TRiP*.*GL00277 sgg RNAi}attP2/+*	118	24,67	2,14	13,68
	*P{TRiP*.*GL00277 sgg RNAi}attP2/Clk 4-1M* Gal4	48	15,83	0	4,46
	*P{TRiP*.*GL00277 sgg RNAi}attP2;35364xtim-*Gal4,*pdf-* Gal80*/+*	113	17,98	0,32	8,99
Over expression of *per*	*w*;*pdf*Gal80; UAS-*per16/*cry-Gal4	61	25,70	1,27	45,28
	*w*;cry-Gal4/+;UAS-*per16*/+	75	25,19	1,49	33,82
	*w;tim*-Gal4/+; UAS-*per16*/+	71	24,98	1,05	35,12
	*w;;*UAS-*per16*/ *Clk 4-1M* Gal4	74	27,1	0,53	7,68
	w;*tim-*Gal4,*pdf*-Gal80/+;UAS-*per16/+*	72	25,33	1,14	44,86
Control animals	*w*^*1118*^*; pdf-* Gal80*/+;cry-*Gal4*/+*	121	25,73	1,09	27,29
	*w*^*1118*^*; cry-*Gal4*/+*	122	25,84	1,01	14,27
	*w*^*1118*^*; tim-*Gal4*/+*	115	26,47	1,17	13,63
	*w*^*1118*^*;; Clk 4-1M* Gal4*/+*	122	25,24	2,02	10,11
	*w*^*1118*^*; tim-*Gal4,*pdf-* Gal80*/+*	118	27,03	0,79	24,09
	*P{EP}sggEP1576*, *w*^*1118*^	125	25,18	0,80	8,97
	*w*^*1118*^*;;UAS-per16/+*	70	23,36	1,52	17,41
	*w*^*1118*^*;;P{UAS-sgg*.*B}MB5/+*	122	24,91	0,43	9,18
	*w*^*1118*^*;; P{TRiP*.*GL00277 sgg RNAi}attP2/+*	114	26,54	0,35	7,94
Positive Control	*cry*^*01*^	109	24,74	0,28	81,96
	*w*^*1118*^	123	26,26	2,39	9,33

Flies (only males) were recorded in LD 12:12 for 7 days and subsequently in LL for at least 14 days. The table displays the percentage of rhythmic flies, the period length and the rhythm of all investigated genotypes in LL according to χ2-periodogram analysis. Furthermore the number of investigated animals is indicated. Animals that died before the end of the experiment were excluded. The table displays a merge of all investigated LL settings, i.e. from LL50 –LL1500. For clarity reasons we did not included the power of the rhythmicity and the genetic background of the animals. This information can be found in Supplementary S1 Table and in S1 Fig. Furthermore the separate data for the different light intensities can be seen in S1 Table.

### Clock Neurons under SGG overexpression

Because western blots with whole heads or our luciferase assays with living animals showed only the signal from the entirety of all clock cells, we wanted to take a closer look at the single clock neurons. Overexpression of SGG under constant light could lead to a stabilization of CRY in only a small subset of clock neurons. Therefore, we used an immunohistochemical approach. Stainings of whole *Drosophila* brains were performed.

**Fig 4:**
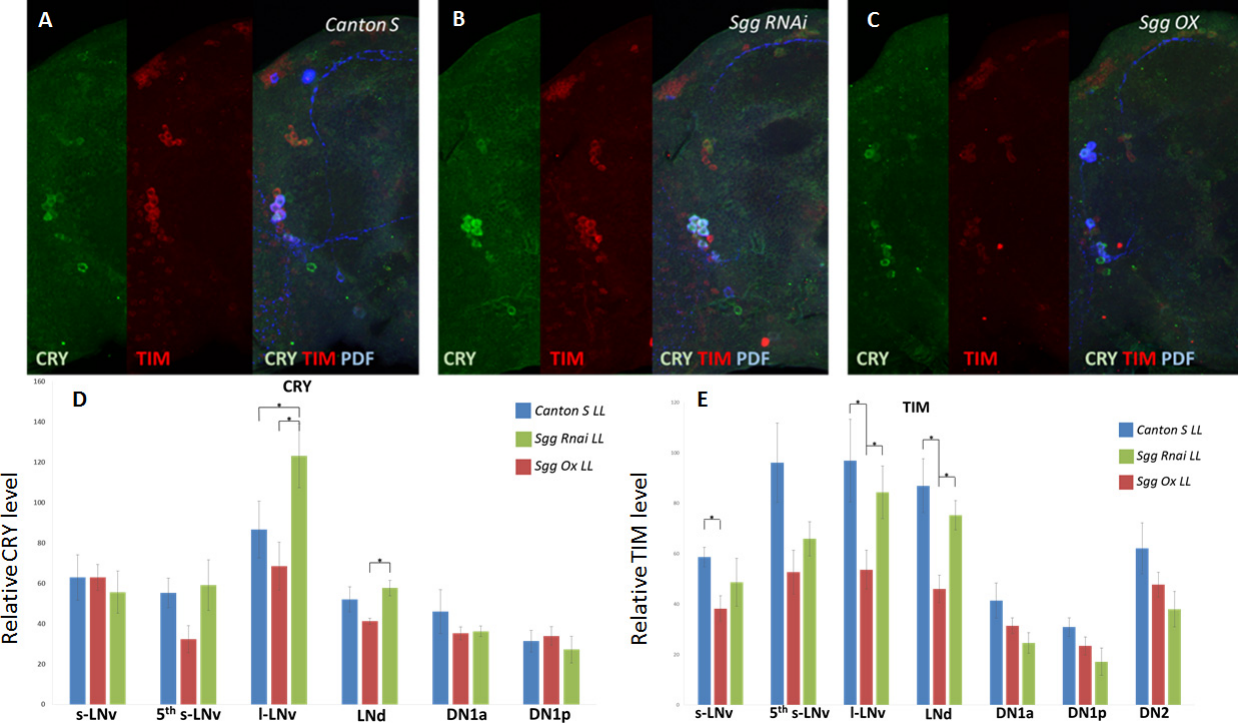
Immunohistochemistry. Whole brains of the indicated genotypes were immunohistochemically investigated with anti-CRY (Yoshii et al. 2008), anti-TIM and anti-PDF antibodies. Animals were investigated on the first day in LL (300 Lux) at timepoint CT 21. (A-C) Representative pictures of the indicated genotypes are shown. (D) and (E) Staining intensity of the clock neurons was measured as described in Material and Methods and quantified. The x-axis displays the different subsets of clock neurons. Brains of at least five animals were averaged. No significant differences in staining intensity were found between *sgg* overexpressing flies and control flies. The only difference was a reduction of TIM in *sgg* overexpressing animals. Data were considered as significantly different at *p<0.05. Significances are indicated by asterisks in the graphs.

The animals were first entrained in LD for three days and then released into LL conditions for 24 hrs. In this experiment, we overexpressed SGG or knocked down SGG by RNAi in all clock neurons. We performed triple stainings with antibodies against CRY, TIM and the Pigment-dispersing factor (PDF). The latter one helps identifying the ventral clock neurons. The staining of the SGG overexpressing animals and the controls did not reveal any clock neuron group in which CRY was stabilized. Quantification of staining intensity did not reveal any significant stabilization effect of SGG overexpression. Our statistical tests revealed a p value of p>0.05. CRY was similar low in all clock cells (Fig 4). The only exception could be seen in the l-LNv neurons. Here our analysis revealed that RNAi mediated knockdown of SGG yielded more CRY, compared to wildtype animals. Furthermore, TIM and CRY expressions were restricted to the cytoplasm, never visible in the cell’s nucleus (Fig 4A–4C). The rhythmically migration of TIM from cytoplasm to the nucleus is important for proper clock function. Because we only investigated a single timepoint in LL conditions, we cannot rule out that a whole series of different timepoints might reveal CRY in the nucleus as well. However, this seems unlikely because Stoleru et al. [21] showed that TIM protein is restricted to the cytoplasm, independent of the investigated timepoint in LL. On the other hand, CRY lacking animals—like *cry*^*b*^–clearly showed nuclear TIM. We conclude that SGG manipulation does neither significantly influence the light mediated degradation of TIM and CRY nor promote nuclear entry of CRY (Fig 4D and Fig 4E). The only significant effect, seen in RNAi mediated knockdown of SGG, would rather argue for a more stable CRY in the absence of SGG, than for a stabilization by SGG overexpression.

### Detecting CRY with different antibodies

Finally, we found that we used a different antibody in our assay compared to Stoleru et al. (2007) [21]. Our polyclonal antibody was raised against full-length dCRY protein (Yoshii et al.) [24], whereas the antibody used in the original publication (Stoleru et al.) [21] was raised against the N-terminal part of dCRY (AA 1–183) fused to a 6-HIS-tag (Rush et al.) [31], raising the possibility that the different antibodies may explain the conflicting results.

**Fig 5:**
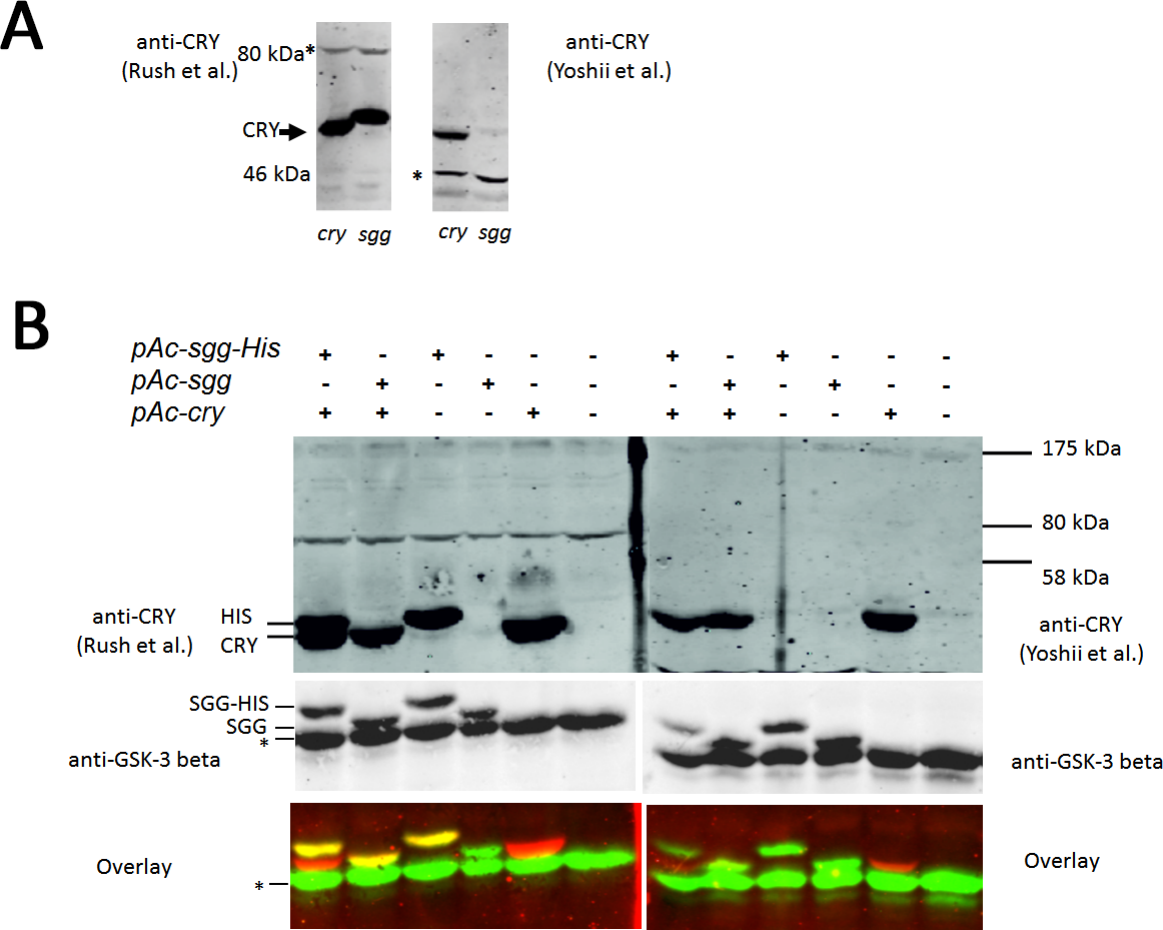
CRY stability in S2 cells–with different antibodies. (A) On the x-axis it is indicated if *pAc-sgg* or *pAc-cry* is expressed. The right western blot was treated with anti-CRY (Yoshii et al.) while for the left western blot another anti-CRY antibody was used (Rush et al.), but apart from the antibodies they were treated the same. The asterisk marks a non-specific band. The cells were kept in darkness. (B) The transfected plasmids are indicated at the top and left, while (+) is transfected, (-) indicates no transfection. Cells were kept in darkness and were released to light before harvesting. The right western blot was treated with anti-CRY (Yoshii et al.) while the left western blot was treated exactly the same, except of another anti-CRY antibody (Rush et al.). In the lower part, the western blot was incubated with anti-GSK-3 antibody. The asterisk marks a non-specific band. In the overlay anti-CRY antibody is coloured red, anti-GSK-3 is green and the overlay of both proteins results in a yellow colour.

To compare the two antibodies we overexpressed *sgg* and *cry* in S2 cells, performed Western Blots and immunostained these in parallel with the two CRY-antibodies. Strikingly, we detected a strong signal in *sgg*-overexpressing flies with the CRY-antibody from Rush et al. [31] but not with the one of Yoshii et al. [24] (Fig 5A). The newly appearing band was running slightly slower, compared to the one detected with our CRY serum (Fig 5A).

There are two possible reasons for that observation. One possibility is that this antibody recognizes a different form of CRY, e.g. a phosphorylated form of this protein, though this does not explain why the faster migrating, presumably unphosphorylated CRY protein is not also visible. If the antibody by Yoshii et al. is not recognizing phosphorylated CRY protein, one would expect to see a strong band in SGG overexpression cells after phosphatase treatment. However, this is not the case (Fig 1C) indicating that the Yoshii et al. antibody recognizes phosphorylated and unphosphorylated CRY. Another possibility to explain the results is that the Rush et al. antibody recognizes the overexpressed SGG protein itself. It is possible that the antibody not only recognizes CRY, but the HIS-tag epitope as well (a HIS-tagged CRY was used for the antibody production–and perhaps the tag was still part of the antigen). Because the overexpressed SGG (the same one as used in Stoleru et al. 2007) [21] was coupled to a HIS-tag, we decided to clone a new *pAc-sgg* vector, where we introduced a stop codon before the following V5 and HIS-tag sequence. Our prediction was that the anti-CRY antibody (Rush et al.) [31] does not recognize this protein anymore. A western blot, where we expressed the SGG protein and the SGG-HIS protein in the presence and absence of CRY confirmed our hypothesis (Fig 5B). While the anti-CRY antibody from Yoshii et al. is recognizing only the overexpressed CRY protein, the antibody from Rush et al. recognizes the SGG-HIS protein, too, but not the SGG protein without a tag. The CRY protein migrates at about 60kDA, while SGG should be detected at about 56kDA.

To determine if the slower migrating band indeed corresponds to SGG, we incubated the western blot with an anti-GSK-3 antibody, which is directed against a region of the catalytic domain of the *Drosophila* GSK-3 enzyme. An overlay of the results, i.e. anti-GSK-3 in green, anti-CRY (Left side Rush et al., right side Yoshii et al.) in red, clearly shows that the anti-CRY (Rush et al.) antibody recognizes SGG-HIS protein in addition to CRY. The discrepancy between the previous study and our own results can therefore be explained by the different specificities of the CRY antibodies applied.

## Discussion

The Glycogen Synthase-kinase 3 beta plays an important role in regulating the circadian clock of different animals, like mice, humans or *Drosophila*. In the latter GSK-3 beta (in *Drosophila* called Shaggy) is directly interacting and phosphorylating master clock proteins like Period [32] or Timeless [18] (and unpublished data). Recently it was reported that another protein, the blue light photoreceptor Cryptochrome, is strongly stabilized by SGG. This stabilization is protecting the animals from constant light arrhythmicity. In our ongoing examination of how exactly this mechanism is working and CRY stabilization leads to LL rhythmicity [21], we encountered the problem that we were not able to reproduce the observed phenotype. When we overexpressed SGG in clock cells we found, that the animals display a period of about 20 hrs in DD conditions [18]. The reason for this faster rhythm is that SGG is phosphorylating PER and TIM, enabling the phosphorylated proteins to enter the cell’s nucleus earlier. The observed changes in behaviour after overexpressing a protein does not necessarily mean that this protein really plays a role under normal, natural conditions. For example, it could be that endogenous SGG is not present in clock cells. SGG mutations are lethal, therefore it is difficult to show the effect of SGG on the clock. It was already published, that expression of a sgg10 cDNA construct under control of a heat shock promoter can rescue the lethality of SGG mutants. Those rescued animals display an elongated period of 26 hrs [18]. However in our experiments, RNAi down-regulation of SGG in the clock cells clearly strengthened the importance of *sgg* for the clock. When we knocked down *sgg* in the ventrally located clock neurons, animals displayed a long period of more than 26 hrs in DD. This new result clearly demonstrates that *sgg* plays an important role in the circadian clock, but that its influence is mainly restricted to the PDF expressing cells and that *sgg’s* role in the Dorsal Neurons (cry-Gal4/pdf-Gal80 or clk 4-1M Gal4) is of minor importance–at least in LD and DD conditions.

The highly phosphorylated form of TIM is more prone to the light induced degradation. Because of this effect, we expected that animals overexpressing SGG might be more sensitive towards light. Consistent with this idea, reducing the activity of SGG leads to a slightly more stable TIM protein and subsequently to animals, which are less sensitive to light [33]. For this reason we were puzzled by the reported apparent opposite effect of SGG on CRY [21]. Because we are not able to reproduce the data we started to doubt that SGG is really directly influencing CRY. We were able to show that the previously reported stabilization effect of SGG on CRY was due to a misinterpretation of data obtained with a CRY antibody that recognizes CRY, but HIS-tagged proteins as well. We believe that the reason for this is found in the production of this antibody—a HIS-tagged CRY was used for the generation. Another awkward coincidence was that CRY and SGG are migrating at a similar size on western blots–explaining why the HIS-tagged SGG was mistakenly recognized as CRY. Next to the stabilization effect it was shown, that CRY is directly interacting with SGG in S2 cells [21]. But because in those experiments the same His-tagged protein was investigated, this interaction is very likely a misinterpretation as well. We were not able to see an interaction of CRY and SGG in S2 cells by CoIP. Stoleru et al. did reveal, that in whole animals SGG can be found in CoIP experiments together with CRY. Because SGG/TIM/PER are directly interacting and CRY is interacting with TIM, this result is not easy to interpret. Therefore we are doubting, that CRY is directly interacting with SGG.

In contrast to previous observations [21], we were not able to elicit strong rhythmic LL behaviour after overexpression of SGG nor to see more light sensitive animals after down regulating *sgg* via RNAi.

The light-sensitivity of flies depends on polymorphisms in *tim* and *jetlag* and on their interaction. We showed that a certain mutation in Jetlag makes the flies less sensitive to light, but only if it is combined with a particular *tim* allele [11]. This clearly demonstrates that the genetic background of the animals is very important. Even more, because the mutation in the *jetlag* gene is lurking in a lot of common laboratory strains. Maybe a similar situation existed in the LL-rhythmic SGG-overexpressing flies [21]. When we took a closer look at the *tim* allele polymorphism and *jetlag* mutation in our investigated experimental strains, we were not able to see a LL rhythm inducing combination of alleles (S3 Fig). But most of the flies analysed in the current study carry the more light-sensitive *s-tim* allele and we were able to find further polymorphisms in the *jetlag* gene. It is possible that the animals in Stoleru et al. carried the more stable *ls-tim* allele and/or common jet[c] allele, resulting in more LL rhythmic animals.

## Materials and Methods

### Fly Strains

Flies were raised on a standard cornmeal/agar medium at 25°C in LD 12:12. For overexpression of *sgg* we used Bloomington strain #11008 *P{EP}sggEP1576 w*^*1118*^ and #5361 *w*^*1118*^*; P{UAS-sgg*.*B}MB5*. For RNAi knockdown we used #35364 *P{TRiP*.*GL00277}attP2*. The driver lines *cry*-Gal4#39 and *tim*-Gal4 are described in[34] and[35].*Clk 4-1M Gal4/TM6B* is described in[27]. *y w; Pdf-*Gal80*/CyO; cry-Gal4/MKRS* was a gift from C. Hermann-Luibl. *w*^*1118*^ is described in[36]. *y w Pdf-* Gal80,*tim-Gal4/CyO* was a gift from R. Stanewsky. *Cry*^*01*^ is described in^[^^37^^]^. *w;;UAS-per16/+* is described in[38]. For the luciferase assay we used in addition *tim-Gal4(62)* as in[39], the *UAS-timeless* lines are described in[40] and *UAS-Luc-dCry* is described in*[13]*. For the wholemount stainings we used *tim(UAS)-Gal4*[35] and *Canton S*[41].

### Behavioural analysis

Locomotor activity of individual flies was recorded using the *Drosophila* Activity Monitoring (DAM) System (Trikinetics) as previously described[42]. We investigated behaviour of 3–7 day old male flies in LD 12:12 for 7 days (with a light intensity of 50, 300 or 1500 lux in the light phase) followed by additional 14 days in constant darkness (DD) or constant light (LL). All recordings took place under constant 25°C in a climate–controlled chamber. Raw data of individual light beam crosses were collected in 1-minute bins and displayed as double-plotted actograms using ActogramJ. This program is a Java plug-in[43] of ImageJ (that can be downloaded at http://rsb.info.nih.gov/ij/). For determining the individual free-running period (τ) of rhythmic flies, DD data from day 2–12 were analysed using χ^2^-periodogram analysis and average period length of each genotype was calculated. Finally, data were averaged across the genotype. For determining the free-running period and rhythmicity, we analysed LL data from day 2–12 in the same way. Only *cry*^*01*^ showed a stable rhythm.

### Cell Culture

The S2 cell line was derived from a primary culture of late stage (20–24 hours old) *Drosophila melanogaster* embryos (Schneider, 1972)[44]. The cells were grown in Insect Xpress medium (Cambrex) with 10% fetal bovine serum (Sigma-Aldrich) and 1% Penicillin-Streptomycin (PAA) at 25°C. Cells were transfected using FectoFly (polyPlus).

*pAc-cry*, *pAc-jet*, *pAc-jet*^*c*^, *pAc-tim* are described in[13]. *pAc-sgg(HIS)* is a gift from P. Nawathean[21]. We used a site-directed Mutagenesis kit (Agilent) to introduce a Stop-Codon for the generation of *pAc-sgg(noHIS)*.

### Western Blots

Cells were harvested 72 hrs after transfection. They were homogenized in protein extraction buffer (20 mM HEPES pH 7.5; 100 mM KCl; 5% glycerol; 10 mM EDTA; 0.1% Triton X-100; 20 mM β-glycerophosphate; 0.1 mM Na3VO4 pH 11) containing a protease inhibitor cocktail (c0mplete Mini EDTA-free; Roche) and loaded onto a 10% gel. For dephosphorylation we treated the protein extract with λ-Phosphatase (Thermo Scientific) for 1 hour. For visualizing the different proteins, we incubated the western blots in primary and secondary fluorescent antibodies with following dilutions: rabbit anti-CRY 1:10000 (kindly provided by T. Yoshii), Alexa Fluor goat-anti-rabbit 680 1:5000 (Invitrogen), rabbit anti-CRY 1:1000 (kindly provided by P. Emery)[31], Mouse Anti-HIS 1:5000 (Invitrogen) Alexa Fluor goat-anti-mouse 1:5000 (Invitrogen), mouse anti-GSK-3 1:5000 (4G-1E; Millipore).

Fluorescent signals were detected using the Odyssey Imaging System (Licor Bioscience).

### Luciferase Assay

Adult flies carrying the luciferase gene fused to dCryptochrome under the control of UAS were fed with luciferin containing food, the resulting bioluminescence measured with a Perkin Elmer TopCount NXT. This assay was performed as in[45] and[13]. For the analysis we averaged the data from 8 different animals.

### Immunohistochemistry

To investigate CRY and TIM stability in adult *Drosophila* brains, 3–7 days old male flies were entrained to LD 12:12 for at least 4 days and then released to constant light conditions. They were sacrificed after 24 hours in light. Flies were fixed in 4% paraformaldehyde (PFA) in 0.1M phosphate buffer (PB; pH 7.4) with 0.1% Triton X-100 for 2.5 hours. The fixation step was carried out on a shaker at room temperature. After dissection 5% normal goat serum (NGS) in PB with 0.5% Triton X-100 was used for blocking samples overnight at 4°C. The brains were incubated with primary antibodies that were diluted in PB with 0.5% Triton X-100, 5% NGS and 0.02% NaN_3_ as follows: rat anti-TIM 1:1000 (kindly provided by I. Edery), mouse anti-PDF 1:1000 (Developmental Studies Hybridoma Bank; DSHB), rabbit anti-CRY 1:1000 (kindly provided by T. Yoshii). After 24–48 hours primary antibody incubation secondary antibodies were applied. Immunolabelings Alexa Fluor 488, Alexa Fluor 555 and Alexa Fluor 647 (all from Molecular Probes) were used as secondary antibodies in a dilution of 1:200 in PB with 5% NGS and 0.5% Triton X-100. After 2 hrs at room temperature secondary antibody solution was removed. Finally, brains were embedded in Vectashield mounting medium (Vector Laboratories). Confocal images were obtained using a Leica TCS SPE confocal microscope. Z-stack images were visualized and edited with the ImageJ distribution Fiji (http://fiji.sc/wiki/index.php/Fiji or http://rsb.info.nih.gov/ij/). Stacks were cropped and compiled as maximum projections. Brightness and contrast were adjusted. For intensity quantification, samples were processed in exactly the same way during the staining protocol and were scanned with identical laser settings. The quantifications were conducted in ImageJ (Fiji). For quantification a square-shaped area of 9 pixels (3 × 3 pixels) was placed on each cell of interest and the average pixel intensity was measured in the brightest focal plane. Cells of at least five different animals were analysed and the intensity values were first background corrected and then averaged for each neuronal group and genotype.

### Statistics

Data were tested for normal distribution applying a onesample Kolmogorov–Smirnov test. To test for significant differences in normally distributed datasets, we then applied a one-way ANOVA followed by a post hoc pairwise comparison with Bonferroni’s correction. Data that were not distributed normally were tested for significant differences with a Kruskal–Wallis test followed by pairwise comparison with Wilcoxon analysis. Data were considered as significantly different at *p<0.05. Significances are indicated by asterisks in the graphs.

### PCR

For genotyping and identifying the timeless *s-tim* and *ls-tim* animals, we used the following oligonucleotide primers to amplify (and later to sequence) the genomic DNA: sense: 5’-TAGGTATCGCCCTCCAAG-3’ and antisense: 5’-TAGGCAGCTCCACAATCA-3’. Sequencing of *jet* gDNA was performed by using oligonucleotides 5`-TGGGATAGAAGTCGTTCAAGT-3`(sense) and 5`-TGCCGATGGCTAACAGAT-3`(antisense) to determine the variants at the common, rare and *jet*^*set*^ [14] sites within two LRR-encoding domains.

## Supporting Information

S1 Fig Overview of the *Drosophila melanogaster* brain, the clock neurons and their arborization. The pictures illustrate the expression patterns of the driver lines that were used in the behavioural experiments. While the GAL4 protein is activating the UAS sequence and thus the following transcript, the GAL80 Protein is inhibiting the transcription of a gene following a UAS sequence. The sophisticated system of activation and repression is working only to a certain extent, so that even in some cells, where GAL80 protein is produced, the inhibition of the UAS transcription might be not 100%.
(TIF)

S2 Fig Behavior of *sgg* manipulated animals in LD conditions. We investigated animals in L(50Lux)D, 25°C, for 7 consecutive days. The graph shows an average of 7 days and at least of 12 animals. The two driver lines *pdf*-Gal80/+;*cry-*Gal4/+ and *tim*-Gal4 were crossed versus wildtype or sgg manipulated animals. While an overexpression or knockdown of *sgg* in the TIM expressing cells leads to a shift of the evening/morning activity peak, the activity after a knockdown of *sgg* is slightly shifted into the night. Expression in the Dorsal neurons or the *white* control did not yield in a change of the activity.
(TIF)

S3 Fig Jetlag and timeless changes in *Drosophila*. When we sequenced our *Drosophila* strains that where investigated in locomotor behaviour, we were able to show that different TIM isoforms were present in the strains. Furthermore we could show that no *jet*^*c*^, *jet*^*r*^ or *jet*^*set*^ mutation is in their *jetlag* gene. But we could see, that a lot more polymorphisms are present in *jetlag*, like L167I or L247V, demonstrating that a test of the proper genetic background is important.
(TIF)

S1 Table Behaviour experiments. (DOCX)
