# Supplementary figures and images for: GSK-3 Beta Does Not Stabilize Cryptochrome in the Circadian Clock of Drosophila
Source: PLoS One. 2016 Jan 7;11(1):e0146571. doi: 10.1371/journal.pone.0146571 (PMC4704813; doi:10.1371/journal.pone.0146571)

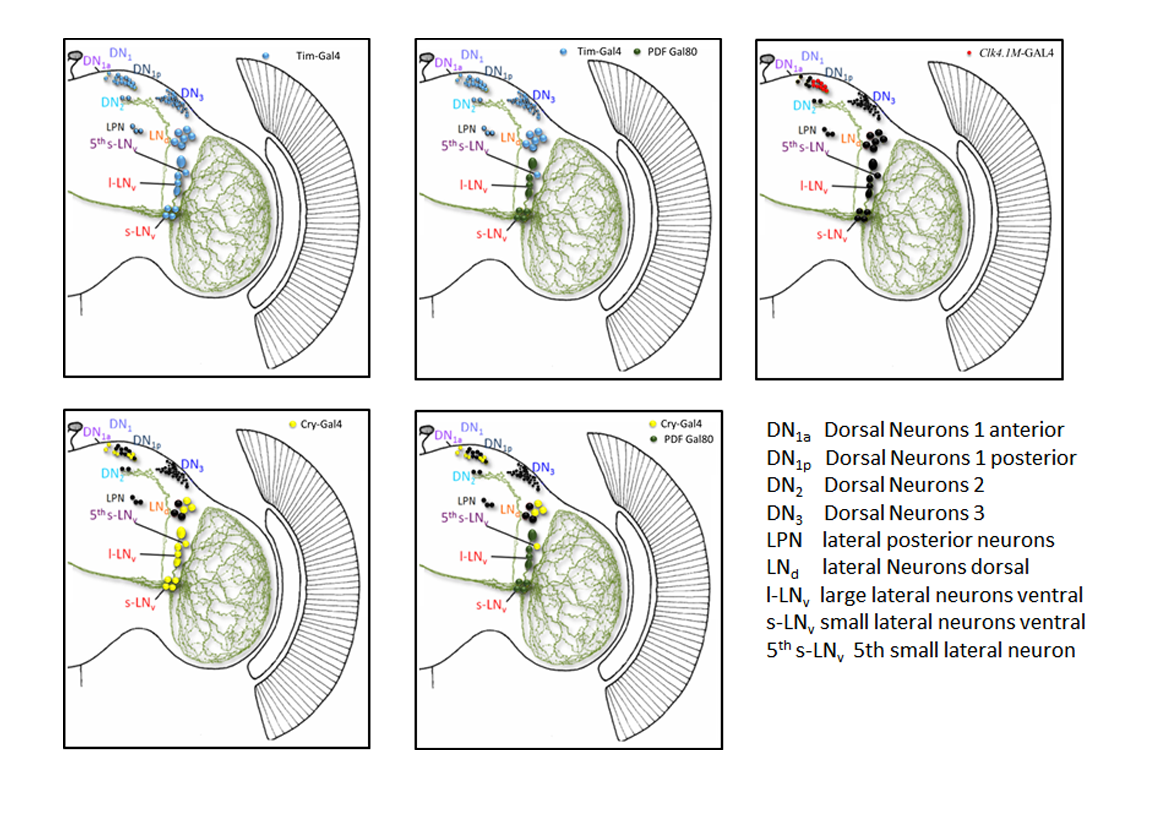

Supplement: S1 Fig — The pictures illustrate the expression patterns of the driver lines that were used in the behavioural experiments. While the GAL4 protein is activating the UAS sequence and thus the following transcript, the GAL80 Protein is inhibiting the transcription of a gene following a UAS sequence. The sophisticated system of activation and repression is working only to a certain extent, so that even in some cells, where GAL80 protein is produced, the inhibition of the UAS transcription might be not 100%. (TIF) [file pone.0146571.s001.tif]

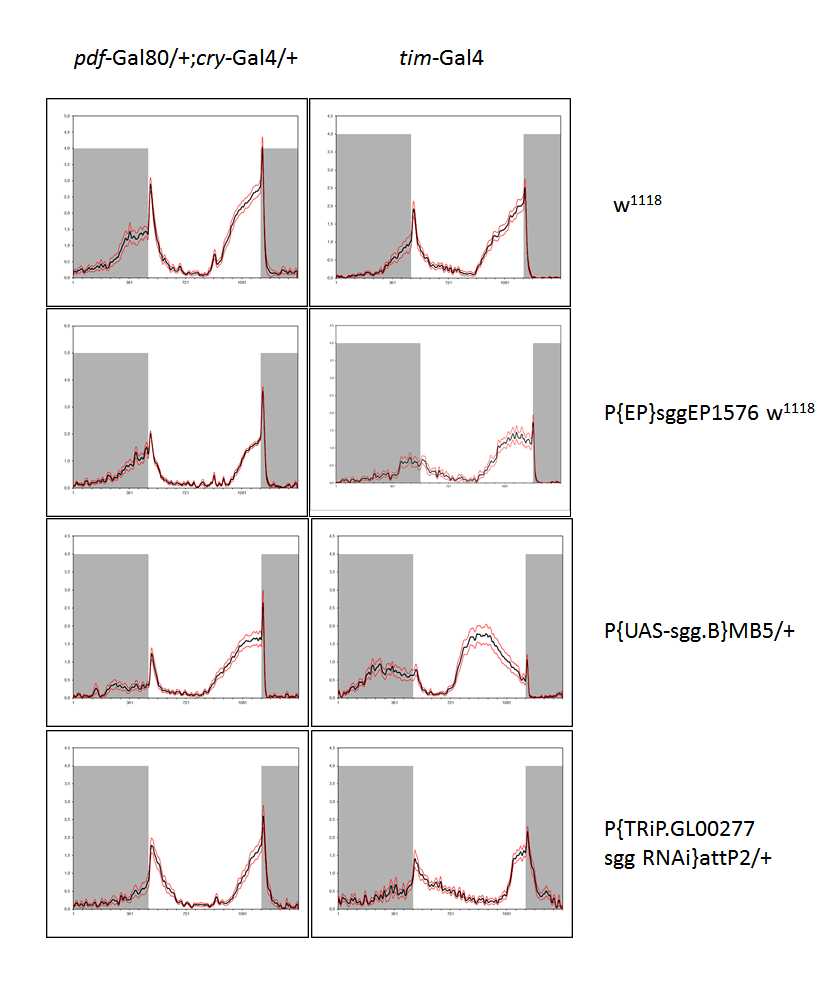

Supplement: S2 Fig — We investigated animals in L(50Lux)D, 25°C, for 7 consecutive days. The graph shows an average of 7 days and at least of 12 animals. The two driver lines pdf-Gal80/+;cry-Gal4/+ and tim-Gal4 were crossed versus wildtype or sgg manipulated animals. While an overexpression or knockdown of sgg in the TIM expressing cells leads to a shift of the evening/morning activity peak, the activity after a knockdown of sgg is slightly shifted into the night. Expression in the Dorsal neurons or the white control did not yield in a change of the activity. (TIF) [file pone.0146571.s002.tif]

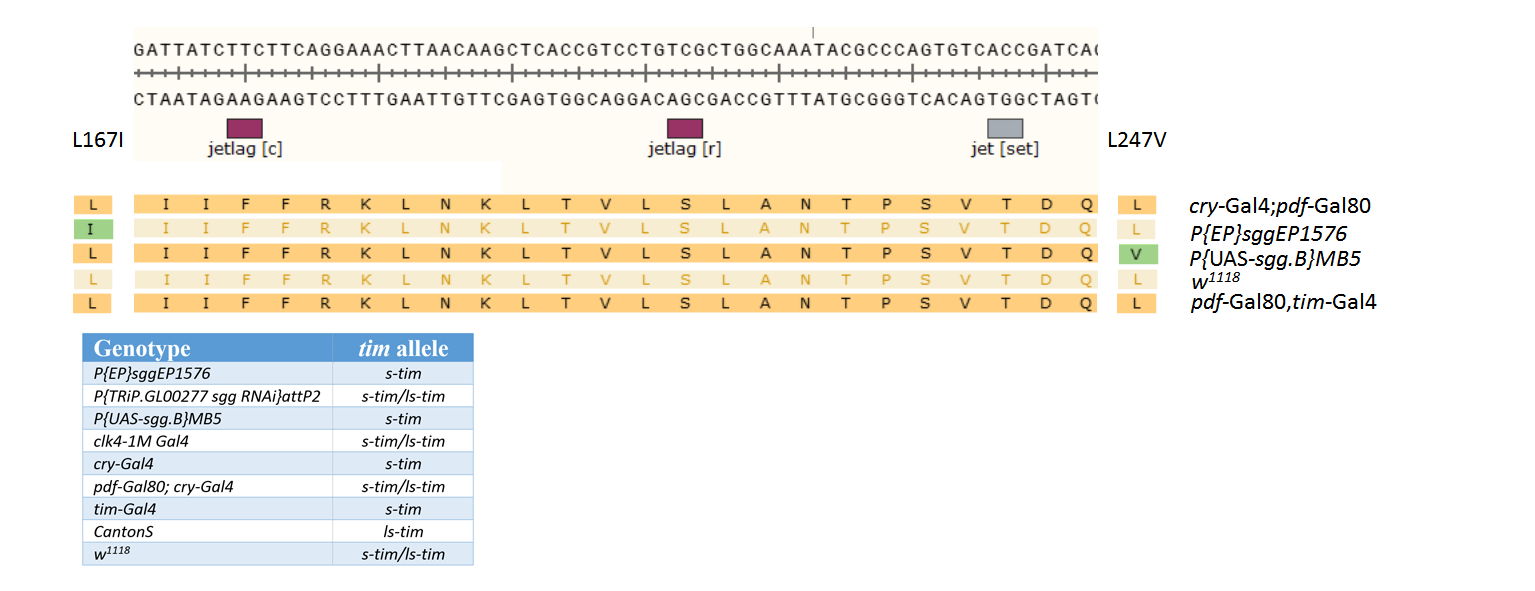

Supplement: S3 Fig — When we sequenced our Drosophila strains that where investigated in locomotor behaviour, we were able to show that different TIM isoforms were present in the strains. Furthermore we could show that no jetc, jetr or jetset mutation is in their jetlag gene. But we could see, that a lot more polymorphisms are present in jetlag, like L167I or L247V, demonstrating that a test of the proper genetic background is important. (TIF) [file pone.0146571.s003.tif]
