# Supplementary material for: GSK-3 Beta Does Not Stabilize Cryptochrome in the Circadian Clock of Drosophila
Source: PLoS One. 2016 Jan 7;11(1):e0146571. doi: 10.1371/journal.pone.0146571 (PMC4704813; doi:10.1371/journal.pone.0146571)
Supplement: S1 Table — (DOCX) [file pone.0146571.s004.docx]

| Group | Genotype | n | period | sem | Rhythmic % | Power | Sem |
| --- | --- | --- | --- | --- | --- | --- | --- |
| Over expression of *sgg* | *P{EP}sggEP1576 w^1118^; Pdf-*Gal80*/+; cry-*Gal4*/+* | 32 | 23,70 | 0,08 | 97 | 53,82 | 2,11 |
|  | *P{EP}sggEP1576 w^1118^; cry-*Gal4*/+* | 27 | 24,91 | 0,18 | 91 | 36,87 | 2,68 |
|  | *P{EP}sggEP1576 w^1118^; tim-*Gal4*/+* | 29 | 21,37 | 0,36 | 76 | 24,38 | 3,00 |
|  | *P{EP}sggEP1576 w^1118^; ; Clk 4-1M* Gal4*/+* | 16 | 24,38 | 0,06 | 100 | 52,3 | 2,52 |
|  | *P{EP}sggEP1576 w^1118^;tim-*Gal4*,pdf-*Gal80*/+* | 14 | 24,83 | 0,13 | 89 | 22,32 | 2,43 |
| Over expression of *sgg* (Uas) | *y w; Pdf-*  Gal80*/+; P{*UAS*-sgg.B}MB5/cry-*Gal*4* | 22 | 23,72 | 0,14 | 83 | 28,63 | 1,29 |
|  | *cry-*Gal4*/+;P{*UAS*-sgg.B}MB5/+* | 29 | 20,94 | 0,14 | 77 | 28,11 | 2,16 |
|  | *tim-*Gal4*/+; P{*UAS*-sgg.B}MB5/+* | 21 | 18,55 | 0,62 | 61 | 28,64 | 1,69 |
|  | *P{*UAS*-sgg.B}MB5/ Clk 4-1M* Gal4 | 16 | 24,16 | 0,07 | 100 | 45,63 | 2,32 |
| Down regulation of *sgg* (RNAi) | *Pdf-*Gal80*/+; P{TRiP.GL00277 sgg RNAi}attP2/cry-*Gal4 | 29 | 24,20 | 0,14 | 93 | 38,77 | 3,48 |
|  | *cry-*Gal4*/+;P{TRiP.GL00277 sgg RNAi}attP2/+* | 28 | 26,21 | 0,15 | 92 | 40,39 | 1,59 |
|  | *tim-*Gal4*/+; P{TRiP.GL00277 sgg RNAi}attP2/+* | 31 | 27,03 | 0,14 | 100 | 43,8 | 2,69 |
|  | *P{TRiP.GL00277 sgg RNAi}attP2/Clk 4-1M* Gal4 | 16 | 24,36 | 0,23 | 100 | 30,89 | 2,87 |
|  | *P{TRiP.GL00277 sgg RNAi}attP2;35364xtim-*Gal4*,pdf-* Gal80*/+* | 16 | 25,29 | 0,13 | 56 | 39,23 | 1,82 |
| Over expression of *per* | *w*;*pdf*Gal80; UAS-*per16/*cry-Gal4 | 13 | 24,18 | 0,04 | 77 | 30,35 | 2,36 |
|  | *w*;cry-Gal4/+;UAS-*per16*/+ | 14 | 23,90 | 0,07 | 57 | 31,13 | 1,63 |
|  | *w;tim*-Gal4/+; UAS-*per16*/+ | 13 | 26,07 | 0,10 | 100 | 47,32 | 2,89 |
|  | *w;;*UAS-*per16*/ *Clk 4-1M* Gal4 | 13 | 24,64 | 0,10 | 92 | 34,23 | 1,43 |
|  | w;*tim-*Gal4,*pdf*-Gal80/+;UAS-*per16/+* | 14 | 24,76 | 0,10 | 57 | 28,56 | 1,8 |
| Control animals | *w^1118^; pdf-* Gal80*/+;cry-*Gal4*/+* | 29 | 23,66 | 0,12 | 97 | 44,53 | 3,62 |
|  | *w^1118^; cry-*Gal4*/+* | 28 | 24,69 | 0,17 | 92 | 32,29 | 2,54 |
|  | *w^1118^; tim-*Gal4*/+* | 29 | 23,84 | 0,11 | 97 | 51,94 | 3,7 |
|  | *w^1118^; ; Clk 4-1M* Gal4*/+* | 32 | 23,90 | 0,11 | 97 | 46,85 | 2,92 |
|  | *w^1118^ ;tim-*Gal4*,pdf-* Gal80*/+* | 14 | 23,61 | 0,17 | 71 | 34,85 | 3,13 |
|  | *P{EP}sggEP1576, w^1118^* | 27 | 24,09 | 0,11 | 87 | 54,44 | 3,0 |
|  | *w^1118^;;UAS-per16/+* | 13 | 23,63 | 0,08 | 92 | 42,16 | 2,54 |
|  | *w^1118^;;P{UAS-sgg.B}MB5/+* | 30 | 23,47 | 0,13 | 93 | 40,08 | 3,07 |
|  | *w^1118^;; P{TRiP.GL00277 sgg RNAi}attP2/+* | 31 | 23,52 | 0,15 | 90 | 42,92 | 2,99 |
| Positiv  Control | *cry^01^* | 29 | 23,07 | 0,08 | 97 | 46,66 | 4,1 |
|  | *w^1118^* | 31 | 23,78 | 0,09 | 93 | 34,96 | 3,25 |

Table S1 A

DD rhythmicity of animals with power values included

| Genotype | n LL50 | period LL50 | sem LL50 | rhythmic LL50 | power | Sem |
| --- | --- | --- | --- | --- | --- | --- |
| *P{EP}sggEP1576 w^1118^; Pdf-*Gal80*/+; cry-*Gal4*/+* | 46 | 28,33 | 0,81 | 13,04 | 19,32 | 2,5 |
| *P{EP}sggEP1576 w^1118^; cry-*Gal4*/+* | 48 | 26 | 1,58 | 20,83 | 19,22 | 2,62 |
| *P{EP}sggEP1576 w^1118^; tim-*Gal4*/+* | 44 | 26,36 | 1,87 | 24,15 | 18,44 | 2,58 |
| *P{EP}sggEP1576 w^1118^; ; Clk 4-1M* Gal4*/+* | 16 | 27,75 | 1,34 | 12,50 | 18,05 | 2,23 |
| *P{EP}sggEP1576 w^1118^;tim-*Gal4*,pdf-*Gal80*/+* | 45 | 23,19 | 1,19 | 49 | 21,12 | 2,72 |
| *y w; Pdf-*  Gal80*/+; P{*UAS*-sgg.B}MB5/cry-*Gal*4* | 39 | 23,83 | 1,33 | 26 | 22,59 | 2,55 |
| *cry-*Gal4*/+;P{*UAS*-sgg.B}MB5/+* | 41 | 24,44 | 1,89 | 26,83 | 21,395 | 2,62 |
| *tim-*Gal4*/+; P{*UAS*-sgg.B}MB5/+* | 36 | 27,30 | 3,32 | 2,78 | 17,12 | 2,58 |
| *P{*UAS*-sgg.B}MB5/ Clk 4-1M* Gal4 | 16 | 0 | 0 | 0 |  |  |
| *Pdf-*Gal80*/+; P{TRiP.GL00277 sgg RNAi}attP2/cry-*Gal4 | 42 | 25,57 | 2,25 | 11,90 | 17,87 | 2,7 |
| *cry-*Gal4*/+;P{TRiP.GL00277 sgg RNAi}attP2/+* | 46 | 25,02 | 0,91 | 21,89 | 16,04 | 3,01 |
| *tim-*Gal4*/+; P{TRiP.GL00277 sgg RNAi}attP2/+* | 43 | 26,07 | 2,08 | 21,55 | 17,55 | 2,29 |
| *P{TRiP.GL00277 sgg RNAi}attP2/Clk 4-1M* Gal4 | 16 | 24,10 | 0 | 7,14 | 16,62 |  |
| *P{TRiP.GL00277 sgg RNAi}attP2;35364xtim-*Gal4*,pdf-* Gal80*/+* | 41 | 26,35 | 0,82 | 17,88 | 17,96 | 2,58 |
| *w*;*pdf*Gal80; UAS-*per16/*cry-Gal4 | 22 | 26,56 | 2,01 | 50 | 23,795 | 3,51 |
| *w*;cry-Gal4/+;UAS-*per16*/+ | 31 | 24,37 | 1,79 | 41,94 | 20,87 | 3,18 |
| *w;tim*-Gal4/+; UAS-*per16*/+ | 27 | 24,11 | 1,14 | 44,44 | 22,39 | 3,51 |
| *w;;*UAS-*per16*/ *Clk 4-1M* Gal4 | 27 | 24,15 | 0 | 7,41 | 20,01 |  |
| w;*tim-*Gal4,*pdf*-Gal80/+;UAS-*per16/+* | 30 | 24,91 | 1,29 | 63,33 | 20,41 | 3,15 |
| *w^1118^; pdf-* Gal80*/+;cry-*Gal4*/+* | 46 | 26,47 | 0,62 | 38,55 | 18,14 | 3,05 |
| *w^1118^; cry-*Gal4*/+* | 46 | 26,49 | 1,40 | 20,50 | 16,44 | 3,02 |
| *w^1118^; tim-*Gal4*/+* | 43 | 27,08 | 1,35 | 13,95 | 18,67 | 2,6 |
| *w^1118^; ; Clk 4-1M* Gal4*/+* | 45 | 28,06 | 2,53 | 16,15 | 17,06 | 2,72 |
| *w^1118^ ;tim-*Gal4*,pdf-* Gal80*/+* | 44 | 26,74 | 0,74 | 38,64 | 22,44 | 3,13 |
| *P{EP}sggEP1576, w^1118^* | 46 | 25,72 | 0,48 | 11,16 | 16,55 | 2,86 |
| *w^1118^;;UAS-per16/+* | 29 | 16,04 | 1,86 | 20,69 | 18,88 | 2,7 |
| *w^1118^;;P{UAS-sgg,B}MB5/+* | 46 | 25,75 | 0,65 | 8,70 | 17,79 | 2,79 |
| *w^1118^;; P{TRiP,GL00277 sgg RNAi}attP2/+* | 40 | 30 | 0 | 2,50 | 17,96 |  |
| *cry^01^* | 42 | 24,21 | 0,46 | 78,21 | 42,94 | 6,16 |
| *w^1118^* | 45 | 24,43 | 1,33 | 11,11 | 18,31 | 2,67 |

Table S1 B

LL rhythmicity of animals investigated under 50 Lux illumination,

| Genotype | n LL300 | period LL300 | sem LL300 | rhythmic LL300 | power | Sem |
| --- | --- | --- | --- | --- | --- | --- |
| *P{EP}sggEP1576 w^1118^; Pdf-*Gal80*/+; cry-*Gal4*/+* | 30 | 27,01 | 1,51 | 16,67 | 18,79 | 4 |
| *P{EP}sggEP1576 w^1118^; cry-*Gal4*/+* | 32 | 25,95 | 2,02 | 12,50 | 18,49 | 4,72 |
| *P{EP}sggEP1576 w^1118^; tim-*Gal4*/+* | 28 | 27,13 | 0,68 | 25 | 19,41 | 4,92 |
| *P{EP}sggEP1576 w^1118^; ; Clk 4-1M* Gal4*/+* | 16 | 22,35 | 1,40 | 13,33 | 18,7 | 5,28 |
| *P{EP}sggEP1576 w^1118^;tim-*Gal4*,pdf-*Gal80*/+* | 32 | 23,48 | 0,86 | 15,63 | 19,64 | 5,08 |
| *y w; Pdf-*  Gal80*/+; P{*UAS*-sgg,B}MB5/cry-*Gal*4* | 22 | 24,54 | 3,91 | 27,27 | 20,33 | 4,48 |
| *cry-*Gal4*/+;P{*UAS*-sgg,B}MB5/+* | 30 | 26,25 | 0,79 | 37,92 | 19,59 | 5,64 |
| *tim-*Gal4*/+; P{*UAS*-sgg,B}MB5/+* | 27 | 26,80 | 0 | 7,16 | 18,52 |  |
| *P{*UAS*-sgg,B}MB5/ Clk 4-1M* Gal4 | 16 | 23,30 | 0 | 6,67 | 16,35 |  |
| *Pdf-*Gal80*/+; P{TRiP,GL00277 sgg RNAi}attP2/cry-*Gal4 | 27 | 26,72 | 0,70 | 14,81 | 18,99 | 4,72 |
| *cry-*Gal4*/+;P{TRiP,GL00277 sgg RNAi}attP2/+* | 32 | 26,23 | 2,05 | 9,38 | 17,96 | 4,08 |
| *tim-*Gal4*/+; P{TRiP,GL00277 sgg RNAi}attP2/+* | 32 | 23,03 | 2,05 | 12,50 | 19,24 | 3,68 |
| *P{TRiP,GL00277 sgg RNAi}attP2/Clk 4-1M* Gal4 | 16 | 23,39 | 0 | 6,25 | 16,21 |  |
| *P{TRiP,GL00277 sgg RNAi}attP2;35364xtim-*Gal4*,pdf-* Gal80*/+* | 28 | 0 | 0 | 0 |  |  |
| *w*;*pdf*Gal80; UAS-*per16/*cry-Gal4 | 15 | 24,87 | 1,07 | 40 | 20,19 | 4,8 |
| *w*;cry-Gal4/+;UAS-*per16*/+ | 14 | 27,43 | 1,42 | 42,86 | 18,71 | 7,04 |
| *w;tim*-Gal4/+; UAS-*per16*/+ | 15 | 26,30 | 0,99 | 33,33 | 19,85 | 4,08 |
| *w;;*UAS-*per16*/ *Clk 4-1M* Gal4 | 15 | 0 | 0 | 0 |  |  |
| w;*tim-*Gal4,*pdf*-Gal80/+;UAS-*per16/+* | 11 | 25,36 | 1,01 | 45,45 | 20,98 | 5,12 |
| *w^1118^; pdf-* Gal80*/+;cry-*Gal4*/+* | 30 | 26,37 | 0,77 | 30 | 20,61 | 5,32 |
| *w^1118^; cry-*Gal4*/+* | 29 | 23,75 | 0,71 | 13,79 | 18,45 | 4,8 |
| *w^1118^; tim-*Gal4*/+* | 28 | 25,14 | 1,06 | 17,86 | 17,1 | 7,24 |
| *w^1118^; ; Clk 4-1M* Gal4*/+* | 31 | 23,47 | 1,56 | 9,68 | 18,25 | 4,88 |
| *w^1118^ ;tim-*Gal4*,pdf-* Gal80*/+* | 30 | 26,25 | 0,61 | 20 | 19,26 | 4,32 |
| *P{EP}sggEP1576, w^1118^* | 32 | 25,93 | 1,92 | 9,38 | 18,54 | 4,04 |
| *w^1118^;;UAS-per16/+* | 15 | 27,10 | 1,89 | 20 | 17,53 | 5,2 |
| *w^1118^;;P{UAS-sgg,B}MB5/+* | 30 | 26,33 | 0 | 16,67 | 15,9 |  |
| *w^1118^;; P{TRiP,GL00277 sgg RNAi}attP2/+* | 31 | 23,57 | 0,74 | 9,68 | 18,75 | 4,44 |
| *cry^01^* | 29 | 25,09 | 0,04 | 93,33 | 39,88 | 16,28 |
| *w^1118^* | 32 | 29,90 | 4,53 | 3,13 | 18,39 | 3,88 |

Table S1 C

LL rhythmicity of animals investigated under 300 Lux illumination

| Genotype | n LL1500 | period LL1500 | sem LL1500 | rhythmic LL1500 | power | Sem |
| --- | --- | --- | --- | --- | --- | --- |
| *P{EP}sggEP1576 w^1118^; Pdf-*Gal80*/+; cry-*Gal4*/+* | 44 | 26,55 | 0,88 | 15,91 | 18,37 | 5,6 |
| *P{EP}sggEP1576 w^1118^; cry-*Gal4*/+* | 44 | 25,96 | 2,96 | 13,64 | 18,26 | 5,28 |
| *P{EP}sggEP1576 w^1118^; tim-*Gal4*/+* | 44 | 24,92 | 1,03 | 18,03 | 19,92 | 5 |
| *P{EP}sggEP1576 w^1118^; ; Clk 4-1M* Gal4*/+* | 16 | 27,29 | 0 | 6,25 | 18,6 |  |
| *P{EP}sggEP1576 w^1118^;tim-*Gal4*,pdf-*Gal80*/+* | 46 | 23,24 | 1,08 | 15,51 | 18,95 | 3,92 |
| *y w; Pdf-*  Gal80*/+; P{*UAS*-sgg,B}MB5/cry-*Gal*4* | 37 | 25,04 | 2,22 | 36,76 | 19,65 | 5,2 |
| *cry-*Gal4*/+;P{*UAS*-sgg,B}MB5/+* | 44 | 25,41 | 2,19 | 26,82 | 18,51 | 5,36 |
| *tim-*Gal4*/+; P{*UAS*-sgg,B}MB5/+* | 41 | 26,77 | 1,62 | 9,59 | 17,88 | 5,16 |
| *P{*UAS*-sgg,B}MB5/ Clk 4-1M* Gal4 | 16 | 0 | 0 | 0 |  |  |
| *Pdf-*Gal80*/+; P{TRiP,GL00277 sgg RNAi}attP2/cry-*Gal4 | 43 | 24,70 | 0,71 | 21,10 | 19,17 | 5,44 |
| *cry-*Gal4*/+;P{TRiP,GL00277 sgg RNAi}attP2/+* | 47 | 24,69 | 1,45 | 17,63 | 17,99 | 5,04 |
| *tim-*Gal4*/+; P{TRiP,GL00277 sgg RNAi}attP2/+* | 43 | 24,90 | 2,30 | 6,98 | 18,31 | 5,04 |
| *P{TRiP,GL00277 sgg RNAi}attP2/Clk 4-1M* Gal4 | 16 | 0 | 0 | 0 |  |  |
| *P{TRiP,GL00277 sgg RNAi}attP2;35364xtim-*Gal4*,pdf-* Gal80*/+* | 44 | 27,60 | 0,13 | 9,09 | 18,3 | 4,08 |
| *w*;*pdf*Gal80; UAS-*per16/*cry-Gal4 | 24 | 25,67 | 0,72 | 45,83 | 21,47 | 6,36 |
| *w*;cry-Gal4/+;UAS-*per16*/+ | 30 | 23,76 | 1,26 | 16,67 | 18,4 | 4,8 |
| *w;tim*-Gal4/+; UAS-*per16*/+ | 29 | 24,52 | 1,02 | 27,59 | 17,14 | 3,84 |
| *w;;*UAS-*per16*/ *Clk 4-1M* Gal4 | 32 | 27,10 | 1,58 | 15,63 | 18,65 | 5,4 |
| w;*tim-*Gal4,*pdf*-Gal80/+;UAS-*per16/+* | 31 | 25,71 | 1,12 | 25,81 | 19,19 | 4,8 |
| *w^1118^; pdf-* Gal80*/+;cry-*Gal4*/+* | 45 | 24,36 | 1,88 | 13,33 | 17,37 | 5,52 |
| *w^1118^; cry-*Gal4*/+* | 47 | 27,30 | 0,92 | 8,51 | 16,53 | 5,68 |
| *w^1118^; tim-*Gal4*/+* | 44 | 27,18 | 1,11 | 9,09 | 17,42 | 5,64 |
| *w^1118^; ; Clk 4-1M* Gal4*/+* | 46 | 24,20 | 1,98 | 4,49 | 17,29 | 5,24 |
| *w^1118^ ;tim-*Gal4*,pdf-* Gal80*/+* | 44 | 28,11 | 1,03 | 13,64 | 18,42 | 3,36 |
| *P{EP}sggEP1576, w^1118^* | 47 | 23,90 | 0 | 6,38 | 16,84 | 4,68 |
| *w^1118^;;UAS-per16/+* | 26 | 26,94 | 0,80 | 11,54 | 17,79 | 4,92 |
| *w^1118^;;P{UAS-sgg,B}MB5/+* | 46 | 22,65 | 0,65 | 2,17 | 17,78 | 3,12 |
| *w^1118^;; P{TRiP,GL00277 sgg RNAi}attP2/+* | 43 | 26,04 | 0,31 | 11,63 | 18,04 | 4,44 |
| *cry^01^* | 38 | 24,93 | 0,33 | 74,34 | 30,88 | 18,72 |
| *w^1118^* | 46 | 24,46 | 1,31 | 13,76 | 17,76 | 5 |

Table S1 D

LL rhythmicity of animals investigated under 1500 Lux illumination
